# Cardiometabolic and Biochemical Indicators in Adolescent Girls According to Nutritional Status and Lifestyle

**DOI:** 10.3390/nu18152500

**Published:** 2026-08-03

**Authors:** Kátia Gianlupi Lopes, Lorraine Aparecida Pinto, Isabela Rezende Ferreira, Arnildo Pott, Rita de Cássia Avellaneda Guimarães, Valter Aragão do Nascimento, Albert Schiaveto de Souza, Giovana Eliza Pegolo, Karine de Cássia Freitas

**Affiliations:** 1 Graduate Program in Health and Development in the Mid-West Region, Federal University of Mato Grosso do Sul, Campo Grande 79070-900, MS, Brazil; kgianlupi@gmail.com (K.G.L.); rita.guimaraes@ufms.br (R.d.C.A.G.); valter.aragao@ufms.br (V.A.d.N.); albert.souza@ufms.br (A.S.d.S.); 2 Prefeitura Municipal de Dourados, Departamento de Atenção Primária, Dourados 79830-220, MS, Brazil; lorrainenutri@hotmail.com; 3 Graduate Program in Nutrition, Federation University of Santa Catarina, Florianópolis 88040-900, SC, Brazil; isabelarf@uol.com.br; 4 Institute of Biosciences, Federal University of Mato Grosso do Sul-UFMS, Campo Grande 79079-900, MS, Brazil; arnildo.pott@gmail.com; 5 Faculty of Pharmaceutical Sciences, Food and Nutrition, Federal University of Mato Grosso do Sul, Campo Grande 79070-900, MS, Brazil; giovana.pegolo@ufms.br

**Keywords:** pediatric obesity, vitamin D deficiency, heart disease, metabolic syndrome, menarche

## Abstract

Background: Adolescence represents an essential stage of life for the prevention of metabolic changes associated with diseases, especially cardiovascular diseases. Objectives: The objectives of this study were to identify nutritional status and associate it with metabolic alterations in adolescent girls. Methods: We conducted a cross-sectional study that included adolescent 10–14-year-old girls from public schools. The research was undertaken with 79 girls. Results: We identified that over half of the girls were sedentary, excessively exposed to screens, physically inactive, and did not practice physical activities in sunlight. The menarche age was significantly lower in girls with overweight and obesity. Most girls skipped breakfast (53.1%) and watched television (84.8%). We observed a high percentage of adolescents with overweight (65.7%), hypovitaminosis D (78.4%) and high levels of triglycerides (69.6%). We did not find an association between hypovitaminosis D and some recorded metabolic parameters (glycemia, glycated hemoglobin, insulin, total cholesterol, HDL-c, LDL-c and triglycerides). However, insulin levels, HOMA-IR, HDL-c, and systolic blood pressure were significantly associated with overweight and obesity, and our results may reflect the characteristics of the study sample and suggest that obese children may already exhibit early signs and risk factors for these chronic diseases during childhood, consistent with national and international data. Conclusions: The identification of metabolic alterations, nutritional status and lifestyle enables early and directed interventions, which may lead to the present and future cardiometabolic health improvements relative to adolescents.

## 1. Introduction

Adolescence represents a phase of physiological, psychological and social changes which cause higher nutritional vulnerability, and the consolidation of unhealthy eating habits can lead to the development of chronic diseases [1], previously considered “adult diseases”, such as diabetes mellitus type 2, hypertension, dyslipidemia, obstructive sleep apnea, non-alcoholic hepatic disease, coronary diseases and various types of cancer, as well as an increased risk of metabolic syndrome (MS) [2].Globally, it is estimated that 507 million children and adolescents, aged 5 to 19 years, will be overweight or obese by 2040 [3]. At the same time, there will also be an increase in the prevalence of comorbidities mediated by insulin resistance, which can develop at an early age due to excess body fat [4].

Cardiovascular health can be preserved through knowledge of most important behavioral risk factors of heart disease and stroke such as poor diet, physical inactivity, smoking and excessive alcohol consumption. These bad habits can cause an increase in blood pressure, raised blood glucose, raised blood lipids, and overweight and obesity [5]. That way some living habits common among adolescents can increase their risk for developing cardiovascular diseases, such as obesogenic diets [6], insufficient sleep [7], physical inactivity and sedentary behavior [8]. In addition, other adolescent behaviors can lead to nutritional deficiencies, e.g., a low intake of nutrients such as calcium [9,10] and vitamin D [11,12,13,14], which can also be related to low sun exposure [15].

There is evidence relating vitamin D shortage and obesity in adolescents [2,16]; this is significantly associated with MS components [17]. The mechanisms of vitamin D’s biological action on MS have not yet been revealed. However, evidence suggests that its insufficiency can alter the function of cell metabolites, including hindering pancreatic function [18] and interfering with the secretion and sensibility of insulin, which represents a central role of MS. In the same way, the vitamin D receptor is present in β pancreatic cells, and adipose and musculoskeletal tissues, among other body tissues, and vitamin D deficiency can jeopardize pro-insulin conversion to insulin, harming glucose utilization and producing glucose intolerance [19]. In addition, vitamin D has an immunomodulatory role and regulates the production of pro-inflammatory cytokines involved in the development process of MS [20]. Even in light of findings that demonstrate the complex relationship between obesity, vitamin D, and metabolic markers in children and adolescents, it is essential to conduct studies aimed at elucidating the mechanisms underlying the role of vitamin D in insulin resistance and obesity [21].

Insulin, a hormone produced by the pancreas, has a fundamental role in respect of glucose entering into effector cells. However, in obese individuals and those with diabetes mellitus type 2, this hormone can accumulate in the blood, leading to insulin resistance. Thus, cells cannot capture excess glucose, causing hyperglycemia and stimulating the production of compensatory insulin. This condition is associated with an increased risk of both obesity and central precocious puberty due to the action of insulin on the hypothalamic–pituitary–adrenal axis and its sensitivity to leptin produced by adipocytes. Thus, the synergism between insulin and leptin seems to be involved in accelerated puberty in obese girls [22]. In turn, particularly among girls, the combination of insulin resistance and vitamin D deficiency has been linked to the pathological mechanisms involved in the onset of central precocious puberty [23]. Finally, girls are at a higher risk of developing juvenile onset of type 2 diabetes than boys, a finding highlighted as a compelling reason to conduct studies on sex differences in glucose metabolism during puberty [24]. Excess adiposity may accentuate the physiological reduction in insulin sensitivity observed during puberty. Longitudinal evidence indicates that adolescents with obesity present lower insulin sensitivity and a greater compensatory insulin response from the early stages of pubertal progression [24]. In Brazilian female adolescents, overweight or obesity and increased waist circumference were associated with insulin resistance, whereas a period of two or more years post-menarche was associated with a lower prevalence of elevated fasting insulin and HOMA-IR [25]. Hyperinsulinemia has also been associated with central precocious puberty because insulin may influence the hypothalamic–pituitary–gonadal axis and interact with leptin produced by adipocytes. Thus, the synergistic effects of insulin and leptin may contribute to accelerated pubertal development in obese girls [26]. This study sheds light on the indicators of nutritional status, lifestyle, and metabolism for a population segment that requires nutritional and health care, particularly with potential applications in the school setting. Metabolic indicators, in particular, provide a more refined understanding of the study sample, especially those characterizing lipid and glycemic profiles and vitamin D levels, thereby lending the study relevance in informing the design of targeted public policies and guiding strategies for the promotion of health and the prevention of health conditions. On the other hand, uncovering aspects related to lifestyle helps raise awareness among the entire school community, families, and relevant agencies regarding the adoption of a care approach grounded in healthy habits, precisely at a stage of life when development is ongoing and can be consolidated throughout adulthood.

The objectives of this study were to assess whether sociodemographic, lifestyle, anthropometric, and metabolic variables are associated with nutritional status, metabolic syndrome, and vitamin D levels among adolescent girls.

## 2. Materials and Methods

### 2.1. Study Population and Sampling Design

The sample was composed of adolescents aged ≥10 to <14 years from 16 urban schools of the School Health Program (SHP) of Dourados (Mato Grosso do Sul-Brazil) in the 2019–2020 cycle. The SHP, created in 2007, is a permanent initiative based on the Health and Education policies for the integral formation of students of basic education in public schools through promotion, prevention and full-care actions of the students [27].

The municipality of our study added 25 schools to the SHP. Given the logistics, we selected only urban schools (n = 16). The age range was defined based on the characteristics of the adolescents in this phase, which involves intense modifications, such as the growth burst period and the pubertal development stage. We also considered the expected menarche and the influence of the participants’ nutritional status.

Initially, we identified girls in the desired age range at each selected public school, and made telephone contact to explain the research and invite them to participate. After acceptance, we performed laboratory tests and interviews at the Health Unit, where we evaluated the girls’ nutritional status and its respective classification. We excluded pregnant and breast-feeding adolescents or those with deficiencies impeding the assessment of weight and height on a portable weighing machine and stadiometer, as well as those whose parents or guardians reported a history of prematurity or low birth weight; the presence of diabetes mellitus; congenital or acquired bone diseases; gastrointestinal diseases followed by bad absorption; nephropathy with or without chronic renal disease; endocrinopathies; cystic fibrosis; celiac disease; use of contraceptives in the 24 months before collection; or the use of drugs that negatively affect bone metabolism (such as anticonvulsants, corticoids and antacids with aluminum) continuously in the last 12 months.

In the sampling, calculation was for convenience. We divided the girls according to their nutritional status into eutrophy, low weight, overweight and obesity; however, we decided to exclude the low-weight group (n = 2) due to its unbalanced number compared with the other groups. Thus, only three groups were left (eutrophy, overweight and obesity).

This project was approved by the Ethics Committee in Research in Human Beings of the Fundação Universidade Federal do Mato Grosso do Sul (UFMS), under the assent n° 5.020.346.

#### 2.1.1. Sociodemographic Variables

For the sociodemographic variables, we applied a form elaborated by their searchers, considering the following: date of birth, sex, self-referred color or ethnicity and economic class. Information about the economic classification was obtained from the Economical Classification Criterion Brazil form [28], filled out by parents or responsible guardians during the interview. The family monthly income ranges corresponding to each economic classification category were as follows: A (USD 4121.23), B1 (USD 2054.96), B2 134 (USD 1089.95), C1 (USD 608.44), C2 (USD 360.94) and DE (USD 164.96).

#### 2.1.2. Behavior and Health Factors Related to Nutritional Status

The behavior variables were gathered via a form and covered the following: daily sun exposure time (over or below 30 min), outdoor physical activity, use of mineral and or vitamin supplements, skipping breakfast, eating in front of screens (television, cell phone, computer or notebook), exposure time to screens (television, cell phone, computer or notebook), smoking, ingestion of alcoholic drinks, intake of matte and soft drinks, and hours of sleep.

To define breakfast, we adopted the criteria utilized by Pereira et al. [29] based on the typical time of the daily first meal of most Brazilians. After determining the time of the participants’ first meal, we standardized it to the previous full hour. We grouped the adolescents who consume food and/or beverages between 6 h and 9 h 59 min as breakfast consumers. To estimate the number of hours of sleep per day, the adolescents stated the time when they go to sleep and wake up on weekdays (Monday to Friday) and weekends (Saturday and Sunday). The average number of daily sleeping hours was calculated by adding the sleeping hours on weekdays multiplied by five, and the sleeping hours on weekend days multiplied by two, then dividing the result by seven [30]. We considered it insufficient to have less than eight hours of sleep a day [31].

#### 2.1.3. Physical Activity Practice and Sedentary Behavior

For physical activity practice, we utilized the Physical Activity Questionnaire for Adolescents (QAFA), an instrument used to measure physical activity in adolescents [32]. This questionnaire was adapted from the study of Sallis et al. [33], and has been tested in adolescents between 14 and 19 years [32]. It was validated for 10–14-year-old adolescents and is considered to have high levels of reproducibility and validity, enabling its utilization for this age range for face-to-face interviews [34].

The adolescents stated the frequency (days/week) and duration (minutes/day) of moderate-to-vigorous-intensity physical activities practiced for at least ten minutes the previous week. The total time of physical activity (minutes/week) was determined by adding the products of frequencies by practice time in each activity. The adolescents practicing below 300 min a week were classified as physically inactive [34]. To measure sedentary behavior, adolescents stated the time spent performing screen activities, such as television, video games, and computers, on weekdays and weekends, using the previous week as a reference period. We calculated the weighted average by adding the average time spent performing screen activities on weekdays multiplied by five, and the average time spent performing screen activities on weekend days multiplied by two, then dividing the result by seven. We defined excessive screen time as spending over two hours a day in such behaviors [35].

#### 2.1.4. Sexual Maturity

For the classification of the sexual maturity stage, we utilized the criteria proposed by Marshall and Tanner [36]. They proposed five levels to classify breast development: M1, M2, M3, M4 and M5. Pubic hair was classified into P1, P2, P3, P4, P5 and P6. Adolescents were considered prepubertal when reported as M1 and P1; pubertal (M2 to M4; P2 to P4) and post-pubertal (M5; P5 and P6). Complementing the evaluation of pubertal development by self-evaluation, the participants reported the age of menarche. Girls who had menarche before 12 years were classified as precocious menarche [37].

#### 2.1.5. Nutritional Status

We evaluated the nutritional status by assessing weight and height, and through analysis of the body mass index (BMI). Body weight was obtained using a Welmy^®^ digital scale (Welmy Indústria e Comércio Ltda, Santa Bárbara d’Oeste—São Paulo, Brazil), with a capacity of 200 Kg and a precision of 100 g. The adolescents removed heavy clothes, adornments, pocket objects and shoes. To measure height, adolescents stayed still, barefoot and fully erect, with the head in a position of the line of sight perpendicular to the body [38]. We utilized a Caumaq^®^ Tape Compact Stadiometer, (Caumaq—Indústria Metalúrgica Ltda, Cachoeira do Sul—Rio Grande do Sul, Brazil), with a capacity of 210 cm. The classification of nutritional status followed the parameters proposed by the World Health Organization (2007) and adopted by the Food and Nutrition Surveillance System [39], which defines the following cut-off points of BMI per age for adolescents: extreme thinness (<score z −3); thinness (≥score z −3 and <score z −2); eutrophy (≥score z −2 and <score z +1); overweight (≥score z +1 and <score z +2); obesity (≥score z +2 and <score z +3) and severe obesity (≥score z +3).

The waist circumference was measured in centimeters, utilizing an inelastic measuring tape, between the last costal arch and the iliac crest, using as a reference the cut-off points indicated by Fernandéz et al. [40]. We measured the neck circumference with the same tape on adolescents standing erect and with the head positioned in the Frankfurt horizontal plane. The upper edge of the tape was placed under the laryngeal salience and perpendicular to the neck long axis, at the thyroid cartilage level, and the circumference was measured with a precision of 0.1 cm. The cut-off point for this measure was 32.7 cm, recommended for girls between 10 and 17 years [41].

#### 2.1.6. Biochemical Evaluation

The biochemical evaluation was performed by a laboratory accredited by the City Council, after 8 h of fasting, through the following analyses: 25 hydroxy-vitamin D, usually indicated as 25 (OH) D; serum calcium; serum phosphorous; parathormone (PTH); insulin; total cholesterol; HDL cholesterol; non-HDL cholesterol; triglycerides; fasting glycemia and glycated hemoglobin.

Vitamin D and insulin were measured using a chemiluminescent immunoassay. The cut-off points for vitamin D levels were as follows: deficiency, 25 (OH) D < 20 ng/mL; insufficiency, 21–29 ng/mL; and sufficient, ≥30 ng/mL. Severe deficiency was considered when values were <10 ng/mL. The girls were classified with hypovitaminosis D when 25 (OH) D < 30 ng/mL [42].

The cut-off point for calcium was 10.1 mg/dL, and for PTH was established as normality within the range 15–65 pg/mL, according to Santos Araújo et al. [43]. Regarding other biochemical parameters, we followed Oliveira [44] for glycated hemoglobin (4 to 6%), serum phosphorous (4.5 to 5.5 mg/dL), serum insulin (<23 µU/mL) and fasting glycemia (75 to 99 mg/dL). The lipogram results were based on the Expert Panel on Integrated Guidelines for Cardiovascular Health and Risk Reduction in Children and Adolescents [45]: total cholesterol: <170 mg/dL; HDL cholesterol: > 45 mg/dL; non-HDL cholesterol: ≥145 mg/dL and triglycerides: <90 mg/dL.

With the values of insulinemia and glycemia, we calculated the Homeostasis Model Assessment-Insulin Resistance Index (HOMA-IR). We utilized the cut-off points indicated by Silva et al. [46]: HOMA-IR > 4.07 to classify insulin resistance in the pubertal phase; in contrast, for girls in the post-pubertal phase, we considered HOMA-IR > 2.91 to predict resistance. For the identification of the frequency of MS, we followed the criteria of the Brazilian Association of Nutrology (ABRAN) [47] for the diagnosis and treatment of MS in children and adolescents, considering the following indicators: excess body adiposity (increased waist circumference); alterations in the lipidic profile (raised triglycerides and or low HDL); altered glycemic profile (high glycemia and or high insulinemia) and high systolic blood pressure (SBP) and/or high diastolic blood pressure (DBP). Thus, adolescents showing three of the four above-mentioned indicators were considered to have MS, according to the cut-off points for girls. The BP was checked with an Omron 705-I^®^ digital device (Omron Healthcare Brasil, Jundiai—São Paulo, Brazil), validated for use on adolescents [48], with a cuff fitting the arm size, while the girl was seated with her feet on the floor, as recommended [49].

#### 2.1.7. Statistical Analyses

The comparison between adolescents with and without hypovitaminosis, or with and without MS, concerning the quantitative variables evaluated in this study, was performed using Student’s *t*-test. Associations between hypovitaminosis D or metabolic syndrome (MS) and categorical variables were evaluated using Pearson’s chi-square test or Fisher’s exact test, as appropriate. For variables involving multiple pairwise comparisons among category levels, *p*-values were adjusted using the Bonferroni correction to control the family-wise error rate. The same analytical approach was applied to examine the associations between nutritional status and the categorical variables included in the study.

The comparison between adolescents with different nutritional statuses concerning the evaluated quantitative variables was performed using a one-way ANOVA followed by Tukey’s post hoc test. The other results were presented in the form of descriptive statistics. The statistical analysis was executed using the statistical program IBM SPSS Statistics for Windows, version 23.0 (IBM Corp., Armonk, NY, USA). A significance level of 5% (*p* < 0.05) was adopted for all analyses. Initially, univariate analyses were conducted to evaluate the associations between the study outcomes and the independent variables. Variables presenting a *p*-value ≤ 0.20 in the univariate analyses were considered eligible for inclusion in the multivariable models.

Binary logistic regression analyses, using the Enter method, were performed to investigate factors associated with hypovitaminosis D and the presence of metabolic syndrome (yes/no). Multinomial logistic regression analyses, also using the Enter method, were performed to identify factors associated with nutritional status categories. All variables selected from the univariate analyses (*p* ≤ 0.20) were entered simultaneously into the corresponding regression model. Adjusted odds ratios (ORs) with their respective 95% confidence intervals (95% CIs) were estimated, and associations with *p* < 0.05 were considered statistically significant.

## 3. Results

We first evaluated 81 adolescents; however, two were excluded for their low weight. Thus, the research was undertaken with 79 girls, with an average age of 139.46 (11.6 years) ± 1.65 months, an average menarche age of 126.15 (10.5 years) ± 1.68 months, most non-white (73.2%) and over half the girls belonging to the income range USD 360.94 to USD 608.44. In Table 1, we show the characterization of the studied sample.

Concerning behavior aspects, 87.3% were sedentary, 85.2% had excessive screen exposure, 64.5% were physically inactive, and 62% did not practice physical activities while exposed to the sun. Regarding eating habits, 53.1% skipped breakfast, 84.8% had meals watching television, 84.8% consumed mate drinks and 13.5% consumed soft drinks regularly.

Regarding nutritional status, 65.7% presented excess weight (overweight, obesity, severe obesity), and 30.3% and 22.7% of adolescents were identified with enlarged waist and neck circumferences, respectively.

Concerning biochemical parameters, the high percentage of hypovitaminosis D (78.4%) and high triglyceride levels (69.6%) stood out, as shown in Table 1.

In Table 2, we show the results of the association between hypovitaminosis D, the variable color/ethnicity, behavior aspects and biochemical tests. Concerning ethnicity, we observed a trend for hypovitaminosis D in white and mixed-race girls (*p* = 0.005); however, the number of black and East Asian adolescents was much lower than other categories, which precludes further inferences. In addition, we observed that adolescents with normal glycemia rates exhibited hypovitaminosis D, i.e., the alteration of glycemia did not relate to alterations in vitamin D values.

Following the univariate analysis of the association between hypovitaminosis D and color/ethnicity, behavioral factors, and biochemical parameters, a multivariable analysis was performed using binary logistic regression. Independent variables with a *p*-value ≤ 0.20 in the univariate analysis (color/ethnicity, physical activity practice, skipping breakfast, glycemia, and HDL cholesterol levels) were included in the model. No significant association was observed between hypovitaminosis D and any of the independent variables included in the multivariable analysis (*p* > 0.05).

Comparing adolescents with different nutritional statuses and the quantitative variables, we noticed a significant difference related to age, menarche age, waist and neck circumference, insulin levels, HOMA-IR, HDL-c and SBP (Table 3). We detected significant differences between the menarche age and the different classifications of nutritional status, especially regarding excess weight (*p* < 0.001). A more precocious menarche age occurred in girls with excess weight (overweight and obesity) than those without excess weight. We also verified a significant association (*p* < 0.001) between adiposity in the abdominal region and neck and nutritional status, which was more significant in girls with obesity. Furthermore, we verified an association between excess weight (overweight and obesity) of adolescents and factors related to cardiovascular risk, such as high insulin and HOMA-IR, low HDL-c and raised SBP.

Following comparison among adolescent girls with different nutritional statuses, considering age, age at menarche, anthropometric and biochemical parameters, and blood pressure, a multivariable analysis was performed using multinomial logistic regression. Independent variables with a *p*-value ≤ 0.20 in the univariate analysis (age, age at menarche, vitamin D, insulin, HOMA-IR, HDL-c, systolic blood pressure [SBP], and diastolic blood pressure [DBP]) were included in the model. No significant association was found between nutritional status and any of the independent variables included in the multivariable analysis (*p* > 0.05).

Table 4 shows the association between nutritional status and lifestyle, behavior, anthropometric, metabolic and sexual maturity variables. We observed that a higher percentage of eutrophic adolescents spent excessive time on screens and had sedentary behavior, and that few had a habit of practicing physical activities while exposed to the sun.

Comparing the nutritional status variables concerning the menarche age, we identified that eutrophic adolescents had a higher average menarche age than adolescents with excess weight (overweight and obesity) (*p* < 0.001). Another result identified was the significant difference between adolescents with different classifications of nutritional status and sexual maturity stages, waist and neck circumference, and plasmatic phosphorous levels. Significant differences in waist and neck circumference (*p* < 0.001) groups of overweight and obesity and the group of eutrophic girls. We also observed that the majority of overweight and obese adolescents did not have excessive screen time, which may be related to lifestyle habits acquired during the pandemic and to post-COVID-19 lifestyle habits, given that schooling has returned to full-time status to make up for missed instructional content.

Following the univariate analysis of the association between nutritional status and lifestyle, behavioral, anthropometric, biochemical, and sexual maturity variables in adolescent girls, a multivariable analysis was performed using multinomial logistic regression. Independent variables with a *p*-value ≤ 0.20 in the univariate analysis (sedentary behavior, physical activity performed under sun exposure, excessive screen time, sexual maturity, waist circumference, neck circumference, phosphorus, calcitonin, insulin, total cholesterol, HDL cholesterol, and triglyceride levels) were included in the model. No significant association was observed between nutritional status and any of the independent variables included in the multivariable analysis (*p* > 0.05).

Table 5 shows the results of comparisons between adolescents with and without MS regarding behavior variables, menarche age, and anthropometric and laboratory parameters. Five adolescent girls were classified with MS. We observed significant differences between groups in respect of the following variables: waist and neck circumference, total cholesterol, HDL cholesterol, non-HDL cholesterol, triglycerides, SBP and DBP. We found the highest values in adolescents with a diagnosis of MS (waist and neck circumference, total cholesterol, non-HDL cholesterol, triglycerides, SBP and DBP) and the lowest HDL-c levels in girls without MS. Supplementary Table S1 presents the results of the association between MS, color/ethnicity, socioeconomic classification, behavior variables, laboratory tests and BP of the evaluated adolescents.

Following comparison between adolescent girls with and without metabolic syndrome, considering behavioral variables, age at menarche, and anthropometric and biochemical parameters, a multivariable analysis was performed using binary logistic regression. Independent variables with a *p*-value ≤ 0.20 in the univariate analysis (age, frequency of sun exposure, daily duration of sun exposure, waist and neck circumferences, vitamin D, insulin, HOMA-IR, total cholesterol, HDL-c, non-HDL cholesterol, and triglyceride levels) were included in the model. No significant association was found between the presence of metabolic syndrome and any of the independent variables included in the multivariable analysis (*p* > 0.05).

## 4. Discussion

The age at which puberty starts has shown a trend of occurring earlier, mainly in girls with high adiposity. There are signs of a reverse association between the BMI and characteristics of pubertal development, such as the thelarche, pubarche and menarche age. Precocious puberty is associated with negative consequences to health, such as depression, cardiovascular diseases, cancer and increased mortality by all causes; hence, it represents a concern for public health [50]. Likewise, Di Sessa et al. [51], evaluating Italian adolescent girls with obesity, verified that 61% developed precocious menarche and presented higher HOMA-IR levels and insulinogenic index.

Our study showed a significant association between nutritional status classes and menarche age, which were 134.40 months (eutrophy), 120.00 months (overweight) and 121.00 months (obesity), once the overweight and obese girls manifested menarche at significantly earlier ages than the eutrophic adolescents. Similarly, Zhou et al. [52] found a strong association between precocious puberty and nutritional status, pointing to 4.67 and 2.22 times the outcome risk for overweight and obese girls, respectively. Obesity in this life phase promotes hyperandrogenism, insulin resistance and compensatory hyperinsulinemia, which can accelerate puberty development. This effect is caused by activation of the hypothalamic–pituitary–gonadal axis or increased bioavailability of sexual hormones due to insulin action in the liver, suprarenal glands, ovaries and adipose tissue [53].

Adipose tissue has also been described as an endocrine organ, producing adipokines, some with pro-inflammatory action and promoting insulin resistance. Obesity hinders insulin signaling, and at the same time, leptin diminishes in the central nervous system. Physiologically, in girls, increased leptin levels occur close to puberty starting, which reach their peak preceding the peak of gonadotropin levels. Leptin serves as a stimulus to the hypothalamic pulse to produce the hormone liberator of gonadotropin, signaling that there is stored energy for female fertility, though not enough to trigger puberty. For that to occur, the interaction of leptin with the neuropeptide kisspeptin is needed. However, testosterone inhibits this neuropeptide, which probably explains the sexual dimorphism of puberty development in girls [37].

Another negative consequence regarding insulin resistance is its central role in the risk of developing diabetes mellitus type 2, and when associated with child obesity, this can trigger metabolic harm linked to the degree of insulin sensitivity. Zhang et al. [54], investigating the long-term effects of obesity in infancy on the risk of developing MS in adults, identified that children with insulin-resistant obesity were 1.7 times more prone to MS in the adult phase than insulin-resistant children with insulin-sensitive obesity. In addition, obesity, metabolic syndrome and insulin resistance in children and adolescents have caused hepatic alterations known as non-alcoholic fatty liver disease, which increases mortality through cardiovascular diseases in adults; hence, the early detection of this liver condition early in life is crucial [55]. Kim and Kim [56], studying Korean adolescents, identified high prevalences of cardiometabolic and MS risk factors according to the severity classification of obesity, i.e., class II and III. Those authors recommend monitoring these risk factors in obese children and adolescents, and especially defining a criterion based on obesity severity.

Another variable evaluated in our study was vitamin D. Nevertheless, despite detecting hypovitaminosis D in 78.4% of the examined adolescents, our study showed that altered glycemia did not relate to alterations in vitamin D levels. Although hypovitaminosis D in adolescents is associated with a worse cardiometabolic profile, particularly central obesity, insulin resistance, higher blood glucose, and lower HDL, most of the evidence is observational, and causality remains uncertain [57]. Furthermore, it is not yet clear how much of this is a direct cause and how much reflects obesity and the environment [58]. Given the multifactorial nature of MetS components, further research is essential to clarify the intricate relationship between them and vitamin D [59].

Among the implications associated with the multifaceted role of vitamin D in health, higher levels of body fat are associated with lower vitamin D levels, as these levels result from dilution in a larger body mass and from storage in adipose tissue. Furthermore, vitamin D deficiency can exacerbate metabolic disorders through inflammation, such as the criteria that define MS [60]. In children and adolescents, low serum levels have also been shown to be associated with an increased risk of obesity; however, longitudinal studies are needed to elucidate the nature of this relationship, which may involve immune regulation, metabolic pathways, and inflammatory signaling [61]. Finally, the pathophysiological relationships between vitamin D, obesity, and MS are still described as complex and underscore the importance of further studies [60]. Regarding behavior variables, our study showed high percentages of sedentary adolescents and excessive exposure to screens (87.83% and 86%, respectively). Delfino et al. [62], investigating the behavior of adolescents, also verified a high use of technological equipment, especially mobile phones/tablets (70%), television and computers (63%), and differences between sexes; girls used mobile phones/tablets more (75.3%) than boys. In addition, the high use of screen devices was associated with a high consumption of fast food, fries and sweets, and physical inactivity.

The health impacts of sedentary behavior at any stage of life have been widely studied. Conversely, particularly among young people, increased light physical activity has been strongly associated with a lower risk of hyperinsulinemia and the progression of insulin resistance from childhood through early adulthood. In turn, an increase in sedentary time has been associated with a worsening of metabolic markers, especially among overweight and obese individuals [63].

In Brazil, the National School Health Survey [64] assessed, among other variables, sedentary behavior and physical activity duration among adolescents aged 13 to 17. The data were collected in 2024 from a representative sample of Brazilian adolescents, and the results indicated that more than one-third of students spent more than two hours a day watching movies, series, soap operas, and other programs on digital video platforms or on television. However, when considering the total time spent commuting between home and school, physical education classes at school, and extracurricular physical activities, 30.6% of adolescents were classified as physically active, while 58.4% were classified as insufficiently active and 9.7% as inactive. Those results allow us to conduct a more comprehensive analysis of the population in question and also corroborate the findings of the present study.

Social distancing advised during the COVID-19 pandemic may have aggravated the harmful habits and lifestyles of adolescents. Malta et al. [65], studying physical activity in Brazilian 12–17-year-old adolescents, verified that the pandemic hindered the practicing of physical activity, dropping from 28.7% to 15.7%, in both sexes and age ranges. The prevalence of sedentary behavior increased in both sexes and age ranges, rising from 44.5% to 70.1%. These characteristics can be observed in the behavior of the adolescent girls included in our study, given that the data were collected during the post-pandemic period and habits acquired during social distancing may have persisted. Thus, regardless of nutritional status, we found high percentages of sedentary adolescents and excessive screen time, which may reflect local population characteristics.

Walking 12,000 steps/day represents a threshold corresponding to the recommendation of 60 min of daily physical activity for adolescents. According to Nagata et al. [66], the combination of less than four hours/day of screen exposure time and the execution of 12,000 steps/day, equivalent to the recommended daily physical activity of moderate to vigorous intensity, was associated with reduced risk of overweight/obesity in adolescents.

Regarding the behaviors of adolescents in our study, we verified high percentages for the intake of mate-based drinks (84.8%) and soft drinks regularly (five times or more per week, 13.5%). The frequent consumption of drinks derived from mate (*Ilex paraguariensis*) is common in South America, such as tea and hot “chimarrão”, and in the state of Mato Grosso do Sul-Brazil, chilled “tereré” is commonly drunk by adolescents and, as a stimulant, can contribute to skipping breakfast. Regarding soft drinks, the 449 ingestion percentages for the adolescents in our study were lower than those in national 450 reports of 30% [67] and 46% [68] the day before the interview. However, this habit is 451 deleterious to an adolescent’s health, increasing the risk of developing overweight and 452 cardiometabolic diseases, mainly due to the excess free sugars [69]. In addition, Atorkey et al. [70] observed that girls consumed soft drinks more often than boys, besides 454 carbonated and alcoholic drinks, which are associated with other health risk behaviors, 455 such as the use of tobacco, consumption of fast food and inadequate intake of fruits and 456 vegetables.

One of the behaviors that is injurious to health and was identified in the examined adolescents is skipping breakfast, confirmed by 53.1% of participants. A systematic review of adolescents from 33 countries verified that skipping breakfast was associated with overweight and obesity in 94.7% of individuals, as well as negative alterations in the lipid profile, blood pressure, insulin resistance and MS [71].

Regarding dietary habits, a study conducted in Brazil involving adolescents aged 12 to 17, drawn from public and private schools across 273 Brazilian municipalities, revealed significant findings concerning food consumption and serum inflammatory biomarkers. According to the authors, this is the only nationwide study of Brazilian adolescents that reflects the dietary culture of this population. The results indicated that the adolescents’ diets exhibited pro-inflammatory characteristics (consumption of soft drinks, cakes, cheese, fats, pasta, snacks, sweets, and bread), particularly among girls. Furthermore, greater adherence to this pro-inflammatory dietary pattern was associated with higher BMI, plasma glucose concentrations, and insulin resistance [72].

Another fundamental aspect complementing the analysis of metabolic implications in adolescents concerns the influence of socioeconomic status. In the present study, we observed a higher prevalence of adolescents from socioeconomic classes C1, C2, and DE, categories representing families with incomes below four times the minimum wage who must carefully manage their resources, particularly when supporting large households. This situation can lead to the routine purchase of nutritionally inadequate foods, such as ultra-processed foods. Furthermore, a study conducted in Brazil among adolescents examines the link between socioeconomic status and metabolic risks, identifying low socioeconomic status as a determinant of insulin resistance [73].

It is important to highlight that in this study, variables such as daily sun exposure time (over or below 30 min), outdoor physical activity, use of mineral and/or vitamin supplements, skipping breakfast, eating in front of screens (television, mobile phone, computer, or laptop), screen exposure time (television, mobile phone, computer, or laptop), smoking, alcohol consumption, intake of mate and soft drinks, and hours of sleep were collected via a questionnaire. This method is one of the most widely used strategies for measuring health-related behaviors, as these instruments are low-cost, easy to administer, and capable of capturing behaviors that would be difficult to observe directly [74].

The veracity and accuracy of these self-reports may be compromised because some health-risk behaviors are difficult to recall, while others are so sensitive that respondents may be reluctant to report them. To minimize such issues, data collection in the present study took place in a private setting [75], with the adolescent left alone. Explanations regarding the importance of the research and guarantees of anonymity, along with a full description of the information to be collected, were provided to ensure truthful responses, as described by Brener et al. [76].

Given the robust evidence of the relationship between the risk for cardiovascular diseases and their origin in the early phases of life, i.e., infancy or adolescence, we highlight that health professionals should approach the primary risk factors in their practice, especially pediatric adiposity, hypertension and hyperlipidemia. Such interventions can result in improvements in reducing the incidence of cardiovascular diseases at the populational level, particularly in adult life, relieving the aftereffects of chronic diseases [71].

## 5. Limitations of the Study

This study has some limitations. Although it was originally planned before the COVID-19 pandemic, the inability to collect data while schools were closed required the adoption of a cross-sectional design and the collection of data immediately after schools reopened. This period was marked by significant changes in the school environment, with the gradual resumption of in-person activities. There was a reduction in sample size, partly due to students migrating from public to private schools, which resumed in-person activities earlier. Furthermore, in order to focus on adolescents enrolled in the School Health Program, one of the study’s eligibility criteria, it was not feasible to include participants from other municipalities, given the limited number of schools participating in that program.

Another significant limitation concerns the sample, which consists exclusively of adolescents from urban schools. Consequently, this characteristic may limit the generalizability of the findings to adolescents in rural and Indigenous schools (with the latter being present in the municipality in question) or to adolescents in schools in other municipalities, due to potential socioeconomic, cultural, and lifestyle differences that could be associated with the outcomes under investigation.

Given the relatively small sample size of this study, particularly the very limited number of participants with metabolic syndrome (n = 5), the stability and reliability of the regression models may be limited. Consequently, the results of these analyses, particularly those from the multinomial logistic regression analyses, should be interpreted with caution and regarded as exploratory rather than conclusive.

Considering the dynamics of classroom instruction, although initially planned, it was not possible to obtain information on dietary intake, as schools prioritized catching up on educational content. Furthermore, the absence of a qualified professional to clinically assess pubertal development made it necessary for participants to self-assess their pubertal stage, a procedure prone to classification errors, even though standardized guidelines were provided to minimize them. Finally, the prolonged period of stay-at-home restrictions resulting from the pandemic may have acted as a confounding factor, as reduced sun exposure may have influenced vitamin D levels regardless of adolescents’ previous lifestyles.

Furthermore, it is worth noting that the use of a questionnaire to estimate physical activity, even if validated, does not provide an objective measure. Consequently, responses may be underestimated or overestimated, in addition to being subject to the influence of the adolescents’ recall. Therefore, conducting studies with instruments that offer greater accuracy should be encouraged among adolescents.

## 6. Conclusions

Our study identified a high percentage of adolescent girls with hypovitaminosis D (78.4%), regardless of nutritional status, as well as high triglyceride levels (68.6%). However, no association was detected between hypovitaminosis D and some biochemical parameters (glycemia, glycated hemoglobin, insulin, total cholesterol, HDL-c, LDL-c and triglycerides).

Concerning behavioral aspects, over half of the girls in our study are sedentary, excessively exposed to screens and physically inactive, and they also do not carry out physical activities while exposed to sunlight. Another relevant result was that more than half (53.1%) of the girls skip breakfast and most (84.8%) eat meals while watching television.

Our study showed that 2/3 of overweight or obese adolescents manifest menarche at earlier ages than eutrophic girls. This result is probably related to the consequences of adiposity on precocious sexual maturity. There are significant differences between adolescents of various classes of nutritional status and sexual maturity stage and waist and neck circumferences, especially in girls with excess weight (overweight and obesity) who have earlier menarche and a wider waist and neck than eutrophic girls. Excess weight is also related to cardiovascular risk; high insulin, HOMA-IR and SBP; and low HDL-c. It is worth emphasizing that hyperinsulinemia is a relevant indicator for the early identification of the risk of developing diabetes mellitus type 2 and other metabolic alterations that will be reflected in the long term in adolescent life.

Regarding the associations between nutritional status and variables related to lifestyle, behavior, anthropometry, biochemistry, and sexual maturation, it is important to interpret the findings with caution; the study’s sample size may have influenced the confirmation of potentially significant results, given that none of the independent variables showed an association with the dependent variables. Nevertheless, the distribution of variable classifications can aid in the design of future studies involving adolescents and highlight the importance of early attention to adolescent health, particularly in cases of excess weight.

Although we identified only five girls diagnosed with metabolic syndrome (MS), we observed significant differences between groups with and without MS and in respect of the following variables: waist and neck circumference, total cholesterol, HDL cholesterol, non-HDL cholesterol, triglycerides, SBP and DBP. We found the highest values in adolescents with a diagnosis of MS (waist and neck circumference, total cholesterol, non-HDL cholesterol, triglycerides, SBP and DBP) and the lowest HDL-c levels in girls without MS.

Considering that several of the metabolic parameters evaluated, especially hypovitaminosis D, were observed in different strata of nutritional status, such findings can be attributed to behavioral factors frequently practiced by adolescents, such as physical inactivity, eating in front of screens, and skipping breakfast. Therefore, it is recommended that the variables investigated in this study be routinely evaluated among all adolescents, regardless of their nutritional status, to identify such changes early and enable interventions that promote the cardiovascular health of these adolescents.

There are significant differences between adolescents of various classes of nutritional status and sexual maturity stage and waist and neck circumferences, especially in girls with excess weight (overweight and obesity) who have earlier menarche and a wider waist and neck than eutrophic girls. Excess weight is also related to cardiovascular risk; high insulin, HOMA-IR and SBP; and low HDL-c. It is worth emphasizing that hyperinsulinemia is a relevant indicator for the early identification of the risk of developing diabetes mellitus type 2 and other metabolic alterations that will be reflected in the long term in adolescent life.

Thus, identifying metabolic alterations, nutritional status, and lifestyle in adolescents can enable early and directed interventions to benefit present and future cardiometabolic health. However, the conduct of longitudinal studies should be encouraged, as they could contribute to a comprehensive understanding and the monitoring over time of conditions associated with excess body fat from early life, given that the present findings describe associations and do not establish temporal or causal relationships.

## Figures and Tables

**Table 1:** Sociodemographic, lifestyle, biochemical and anthropometric characteristics of the sample.

Variable	% (n)	95% CI (%)
Color/ethnicity		
Mixed Race	68.3 (54)	56.8–78.3
White	26.5 (21)	17.3–37.9
Black	3.7 (3)	0.8–10.7
East Asian	1.2 (1)	0.03–6.9
Economic classification		
A	1.2 (1)	0.03–6.9
B1	5.0 (4)	1.4–12.5
B2	16.4 (13)	9.0–26.2
C1	36.7 (29)	26.1–48.3
C2	26.5 (21)	17.3–37.9
DE	13.9 (11)	7.2–23.5
Sedentary behavior		
No	12.6 (10)	6.2–21.8
Yes	87.3 (69)	78.2–93.8
Physical activity		
Physically inactive	64.5 (51)	52.9–75.0
Physically active	35.4 (28)	25.0–47.1
Sun exposure		
No	44.3 (35)	33.1–56.0
Yes	55.6 (44)	44.0–66.9
Physical activity practice outdoors		
No	62.0 (49)	50.4–72.7
Yes	37.9 (30)	27.3–49.6
Use of mate-based drinks		
No	15.1 (12)	8.1–25.0
Yes	84.8 (67)	75.0–91.9
Regular ingestion of soft drinks		
No	86.0 (68)	76.5–92.8
Yes	13.6 (11)	7.2–23.5
Skipping breakfast		
No	46.8 (37)	35.5–58.4
Yes	53.1 (42)	41.6–64.5
Excessive screen time		
No	13.9 (11)	7.2–23.5
Yes	86.0 (68)	76.5–92.8
Eating in front of TV		
No	15.1 (12)	8.1–25.0
Yes	84.8 (67)	75.0–91.9
Sexual maturity		
Prepubertal	5.0 (4)	1.4–12.5
Pubertal	82.2 (65)	71.8–89.7
Postpubertal	12.6 (10)	6.2–21.8
Nutritional status		
Eutrophy	34.1 (27)	23.8–45.7
Overweight	32.9 (26)	22.8–44.3
Obesity	32.9 (26)	22.8–44.3
Waist circumference		
Normal	69.6 (55)	58.2–79.4
Enlarged	30.3 (24)	20.6–41.8
Neck circumference		
Normal	77.2 (61)	66.4–85.9
Enlarged	22.7 (18)	14.1–33.6
Vitamin D		
Without hypovitaminosis	12.6 (10)	6.2–21.8
Hypovitaminosis	78.4 (62)	67.8–87.1
Without information	8.8 (7)	3.6–17.2
Insulin levels		
Normal	75.9 (60)	64.8–84.9
High	13.9 (11)	7.2–23.5
Without information	10.1 (8)	4.5–18.9
Total cholesterol levels		
Normal	63.2 (50)	51.6–73.7
High	32.9 (26)	22.8–44.3
Without information	3.8 (3)	0.8–10.7
HDL levels		
Normal	77.2 (61)	66.4–85.9
Altered	18.9 (15)	11.0–29.1
Without information	3.8 (3)	0.8–10.7
Triglyceride levels		
Normal	27.8 (22)	18.3–39.1
High	69.6 (55)	58.2–79.4
Without information	2.5 (2)	0.3–8.8

**Table 2:** Association between hypovitaminosis D, the variable color/ethnicity, behavior aspects and biochemical tests in adolescents.

Variable	Hypovitaminosis D		*p*-Value
	No	Yes	
Color/ethnicity			
Mixed Race	10.0 (2) ^a^	90.0 (18) ^a^	0.005
White	12.0 (6) ^a^	88.0 (44) ^a^	
Black	66.7 (2) ^a^	33.3 (1) ^a^	
East Asian	100.0 (1) ^a^	0 (0) ^a^	
Sedentary behavior			
No	11.1 (1)	88.9 (8)	1.000
Yes	15.4 (10)	84.6 (55)	
Physical activity practice			
No	10.4 (5)	89.6 (43)	0.144
Yes	23.1 (6)	76.9 (20)	
Sun exposure			
No	13.9 (5)	86.1 (31)	0.818
Yes	15.8 (6)	84.2 (32)	
Physical activity practice outdoors			
No	14.9 (7)	85.1 (40)	1.000
Yes	14.8 (4)	85.2 (23)	
Use of supplement			
No	14.7 (10)	85.3 (58)	1.000
Yes	16.7 (1)	83.3 (5)	
Skipping breakfast			
No	21.1 (8)	78.9 (30)	0.192
Yes	8.3 (3)	91.7 (33)	
Excessive screen time			
No	18.2 (2)	81.8 (9)	0.664
Yes	14.3 (9)	85.7 (54)	
Eating in front of TV			
No	28.6 (4)	71.4 (10)	0.203
Yes	11.7 (7)	88.3 (53)	
Insulin levels			
Normal	15.5 (9)	84.5 (49)	1.000
High	9.1 (1)	90.9 (10)	
Glycemia rates			
Normal	13.7 (10)	86.3 (63)	0.149
High	100.0 (1)	0.0 (0)	
Glycated hemoglobin levels			
Normal	6.2 (11)	83.8 (57)	1.000
High	0.0 (0)	100.0 (1)	
Total cholesterol levels			
Normal	14.3 (7)	85.7 (42)	1.000
High	16.0 (4)	84.0 (21)	
HDL levels			
Normal	18.0 (11)	82.0 (50)	0.194
Altered	0.0 (0)	100.0 (13)	
Levels of triglycerides			
Normal	8.7 (2)	91.3 (21)	0.485
High	17.6 (9)	82.4 (42)	

The results are presented as relative frequency (absolute frequency). *p*-value in the Chi-squared test or Fisher’s exact test when appropriate. ^a^ Identical letters within the column indicate no significant differences in hypovitaminosis D among girls of different color/ethnicity categories (Chi-square test with Bonferroni correction, *p* > 0.05).

**Table 3:** Comparison between adolescent girls with different nutritional statuses, considering the variables age, menarche age, anthropometric and biochemical parameters and blood pressure.

Variable	Nutritional Status			*p*-Value	η^2^	Magnitude
	Eutrophy	Overweight	Obesity			
Age (months)	146.89 ± 3.25 ^a^	135.23 ± 2.48 ^b^	135.46 ± 2.32 ^b^	0.004	0.137	Large
Menarche age (months)	134.40 ± 2.09 ^a^	120.00 ± 1.92 ^b^	121.00 ± 2.75 ^b^	<0.001	0.422	Large
Frequency of sun exposure * (number of days/week)	3.73 ± 0.43	3.88 ± 0.35	4.31 ± 0.33	0.514	0.032	Small
Amount of sleep/day ** (hours)	494.44 ± 20.38	520.38 ± 17.29	498.85 ± 13.44	0.531	0.016	Small
Calcium (mg/dL)	9.58 ± 0.10	9.18 ± 0.10	9.14 ± 0.36	0.278	0.035	Small
Phosphorous (mg/dL)	4.91 ± 0.17	5.40 ± 0.36	4.97 ± 0.14	0.296	0.034	Small
Vitamin D (ng/mL)	23.56 ± 1.29	20.28 ± 1.25	21.77 ± 1.32	0.200	0.046	Medium
Parathormone (pg/mL)	35.22 ± 3.21	35.01 ± 2.43	51.30 ± 12.19	0.201	0.046	Medium
Insulin (µU/mL)	10.49 ± 1.43 ^b^	12.06 ± 1.42 ^b^	21.04 ± 3.13 ^a^	0.002	0.169	Large
HOMA-IR	2.22 ± 0.26 ^b^	2.58 ± 0.34 ^b^	4.38 ± 0.66 ^a^	0.003	0.157	Large
Glycemia (mg/dL)	85.04 ± 1.50	84.35 ± 1.42	84.86 ± 1.29	0.938	0.002	Small
Glycated hemoglobin (%)	5.24 ± 0.05	5.29 ± 0.11	5.26 ± 0.08	0.912	0.003	Small
Total cholesterol (mg/dL)	156.00 ± 5.25	152.54 ± 4.86	159.92 ± 5.14	0.605	0.014	Small
HDL-c (mg/dL)	54.63 ± 1.48 ^a^	49.29 ± 1.63 ^ab^	48.00 ± 1.68 ^b^	0.009	0.121	Large
Non-HDL cholesterol (mg/dL)	101.37 ± 5.47	103.25 ± 4.56	110.88 ± 5.15	0.382	0.026	Small
Triglycerides (mg/dL)	116.37 ± 10.00	97.79 ± 9.49	118.84 ± 9.47	0.262	0.036	Small
SBP (mmHg)	94.81 ± 2.20 ^b^	106.19 ± 2.41 ^a^	113.50 ± 2.67 ^a^	<0.001	0.285	Large
DBP (mmHg)	65.07 ± 1.32	69.73 ± 1.93	71.23 ± 3.05	0.123	0.054	Medium

* Sun exposure: number of days/week; ** Amount of sleep/day: minutes. The results are presented as the mean ± standard error. *p*-value in a one-way ANOVA. Different letters in the line indicate significant differences between girls with different nutritional statuses (Tukey’s post hoc test, *p* < 0.05). SBP: systolic blood pressure, DBP: diastolic blood pressure.

**Table 4:** Association between nutritional status and lifestyle, behavior, anthropometric, biochemical and sexual maturity variables in adolescent girls.

Variable	Nutritional Status			*p*-Value
	Eutrophic (%)	Overweight (%)	Obesity (%)	
Sedentary behavior				
No	0.0 (0)	50.0 (5)	50.0 (5)	0.051
Yes	39.1 (27)	30.4 (21)	30.4 (21)	
Physical activity practice in the sun				
No	44.9 (22) ^a^	24.5 (12) ^a^	30.6 (15) ^a^	0.036
Yes	17.2 (5) ^b^	44.8 (13) ^a^	37.9 (11) ^a^	
Excessive screen time				
No	0.0 (0) ^b^	45.5 (5) ^a^	54.5 (6) ^a^	0.033
Yes	39.7 (27) ^a^	30.9 (21) ^a^	29.4 (20) ^a^	
Sexual maturity				
Prepubertal	25.0 (1) ^ab^	75.0 (3) ^a^	0.0 (0) ^a^	
Pubertal	29.2 (19) ^b^	33.8 (22) ^ab^	36.9 (24) ^a^	0.037
Postpubertal	70.0 (7) ^a^	10.0 (1) ^b^	20.0 (2) ^a^	
Waist circumference				
Normal	49.1 (27) ^a^	41.8 (23) ^a^	9.1 (5) ^b^	<0.001
Enlarged	0.0 (0) ^b^	12.5 (3) ^b^	87.5 (21) ^a^	
Neck circumference				
Normal	42.6 (26) ^a^	37.7 (23) ^a^	19.7 (12) ^b^	<0.001
Enlarged	5.6 (1) ^b^	16.7 (3) ^a^	77.8 (14) ^a^	
Smoking				
No	34.6 (27)	32.1 (25)	33.3 (26)	0.356
Yes	0.0 (0)	100.0 (1)	0.0 (0)	
Consumption of alcoholic drinks				
No	34.2 (27)	32.9 (26)	32.9 (26)	-
Yes	0.0 (0)	0.0 (0)	0.0 (0)	
Consumption of mate-based drinks				
No	33.3 (4)	25.0 (3)	41.7 (5)	0.740
Yes	34.3 (23)	34.3 (23)	31.3 (21)	
History of fractures				
No	35.9 (23)	34.4 (22)	29.7 (19)	0.452
Yes	26.7 (4)	26.7 (4)	46.7 (7)	
Phosphorous levels				
Normal	26.0 (13) ^b^	42.0 (21) ^a^	32.0 (16) ^a^	0.018
Altered	54.2 (13) ^a^	12.5 (3) ^b^	33.3 (8) ^a^	
Parathormone levels				
Normal	22.8 (22)	35.4 (23)	30.8 (20)	0.550
Altered	33.3 (2)	16.7 (1)	50.0 (3)	
Calcitonin levels				
Normal	64.0 (16)	20.0 (5)	16.0 (4)	0.177
Altered	0.0 (0)	100.0 (1)	0.0 (0)	
Insulin levels				
Normal	35.0 (21)	36.7 (22)	28.3 (17)	0.075
Elevated	18.2 (2)	18.2 (2)	63.6 (7)	
Glycemia				
Normal	34.7 (26)	32.0 (24)	33.3 (25)	0.399
Elevated	100.0 (1)	0.0 (0)	0.0 (0)	
Glycated hemoglobin levels				
Normal	37.1 (26)	31.4 (22)	31.4 (22)	0.347
Elevated	0.0 (0)	100.0 (1)	0.0 (0)	
Total cholesterol levels				
Normal	38.0 (19)	36.0 (18)	26.0 (13)	0.195
Elevated	30.8 (8)	23.1 (6)	46.2 (12)	
HDL levels				
Normal	41.0 (25)	29.5 (18)	29.5 (18)	0.129
Elevated	13.3 (2)	40.0 (6)	46.7 (7)	
Triglyceride levels				
Normal	18.2 (4)	50.0 (11)	31.8 (7)	0.066
Elevated	41.8 (23)	25.5 (14)	32.7 (18)	

The results are presented as relative frequency (absolute frequency). *p*-value in the Chi-squared test. Different letters in the column indicate significant differences between girls with different sexual maturity stages, classification of waist and neck circumference, and different phosphorous rates concerning nutritional status (Chi-squared test with Bonferroni correction, *p* < 0.05).

**Table 5:** Comparison between adolescents with and without metabolic syndrome considering behavior variables, menarche age, and anthropometric and biochemical parameters evaluated in this study.

Variable	Metabolic Syndrome		*p*-Value
	No	Yes	
Age (months)	140.25 ± 1.69	129.50 ± 5.97	0.088
Menarche age (months)	126.15 ± 10.97	126.00 ± 6.00	0.985
Frequency of sun exposure (days/week)	3.87 ± 0.21	5.00 ± 0.63	0.085
Amount of sleep/day (minutes)	507.73 ± 10.13	460.00 ± 3.924	0.188
Waist circumference (cm)	72.09 ± 1.24	97.58 ± 3.98	<0.001
Neck circumference (cm)	30.97 ± 0.26	36.08 ± 1.49	<0.001
Calcium (mg/dL)	9.42 ± 0.07	7.88 ± 1.65	0.403
Phosphorous (mg/dL)	5.13 ± 0.15	4.54 ± 0.14	0.293
Vitamin D (ng/mL)	21.85 ± 0.77	26.35 ± 3.42	0.108
Parathormone (pg/mL)	40.25 ± 4.33	34.48 ± 6.93	0.751
Insulin (µU/mL	13.10 ± 1.11	27.67 ± 9.58	0.190
HOMA-IR	2.75 ± 0.24	5.83 ± 1.92	0.170
Glycemia (mg/dL)	84.51 ± 0.84	86.75 ± 2.02	0.452
Glycated hemoglobin (%)	5.24 ± 0.05	5.44 ± 0.14	0.287
Total cholesterol (mg/dL)	153.85 ± 3.04	177.33 ± 5.15	0.031
HDL-c (mg/dL)	51.50 ± 0.95	42.33 ± 2.75	0.009
Non-HDL cholesterol (mg/dL)	101.99 ± 2.98	135.00 ± 4.45	0.002
Triglycerides (mg/dL)	105.13 ± 5.39	170.50 ± 20.28	0.001

The results are presented as the mean ± standard error of the mean. *p*-value in Student’s *t*-test.

## Data Availability

The original contributions presented in the study are included in the article; further inquiries can be directed to the corresponding author.

## Supplementary Materials

The following supporting information can be downloaded at: https://www.mdpi.com/article/10.3390/nu18152500/s1, Table S1: Association between metabolic syndrome, color/ethnicity, economic classification, behavior variables, laboratory exams and blood pressure of the evaluated adolescents.

## Abbreviations

The following abbreviations are used in this manuscript:

BMI	Body mass index
DBP	Diastolic blood pressure
HDL-c	High-density lipoprotein cholesterol
HOMA-IR	Homeostatic model assessment of insulin resistance
LDL-c	Low-density lipoprotein cholesterol.
MS	Metabolic syndrome
PTH	Parathormone
SBP	Systolic blood pressure
