# Supplementary material for: Cardiometabolic and Biochemical Indicators in Adolescent Girls According to Nutritional Status and Lifestyle
Source: Nutrients. 2026 Aug 3;18(15):2500. doi: 10.3390/nu18152500 (PMC13467703; doi:10.3390/nu18152500)
Supplement: Supplementary file 1 [file nutrients-18-02500-s001.zip › nutrients-4425623-supplementary.pdf]

nutrients

MDPI

Supplementary Materials:

Supplemantary Table:

Table S1. Association between metabolic syndrome, color/ethnicity, economic classification, behavior variables, laboratory exams and blood pressure of the evaluated adolescents.

**Variables**	Metabolic Syndrome		**P value**
	**No**	**Yes**	
**Color/ethnicity**			
Mixed Race	69.6 (48)	66.7 (6)	0.85[(b)]
White	24.6 (17)	33.3 (3)	
Black[(d)]	4.3 (3)	0.0 (0)	
East Asian[(c)]	1.5 (1)	0.0 (0)	
**Economic classification**			
A (USD 4121.23)	1.5 (1)	0.0 (0)	0.30[(b)]
B1(USD 2054.96)	4.3 (3)	11.1 (1)	
B2 (USD 1089.85)	17.4 (12)	11.1 (1)	
C1 (USD 608.44)	31.9 (22)	66.7 (6)	
C2 (USD 360.94)	29.0 (20)	11.1 (1)	
DE (USD 164.26)	15.9 (11)	0.0 (0)	
**Sedentary behavior**			
No	11.6 (8)	22.2 (2)	0.32[(a)]
Yes	88.4(61)	77.8 (7)	

nutrients

MDPI

**Physical activity classification**0.49[a]			
Physically inactive	63.8 (44)	77.8 (7)	0.49[a]
Physically active	36.2 (25)	22.2 (2)	
**Sun exposure**			
No	47.8 (33)	22.2 (2)	0.18[a]
Yes	52.2 (36)	77.8 (7)	
**Physical activity practice in the sun**			
No	63.8 (44)	55.6 (5)	0.72[a]
Yes	36.2 (25)	44.4 (4)	
**Use of supplement**			
No	92.7 (64)	88.9 (8)	0.53[a]
Yes	7.3 (5)	11.1 (1)	
**Skipping breakfast**			
No	47.8 (33)	44.4(4)	1.00[a]
Yes	52.2 (36)	55.6(5)	
**Excessive screen time**			
No	13.0 (9)	22.2 (2)	0.61[a]
Yes	87.0 (60)	77.8 (7)	
**Eating in front of TV**			
No	17.4(12)	0.0 (0)	0.34[a]
Yes	82.6(57)	100.0(9)	
**Smoking**			
No	98.5 (68)	100.0(9)	1.00[a]
Yes	1.5 (1)	0.0 (0)	
**Consumption of alcoholic drinks**			
No	100.0 (69)	100.0(9)	-
Yes	0.0 (0)	0.0 (0)	

**Consumption of mate-based drinks**			
No	11.6 (8)	44.4 (4)	0.03(a)
Yes	88.4 (61)	55.6 (5)	
**History of fractures**			
No	81.2 (56)	77.8 (7)	1.00(a)
Yes	18.8 (13)	22.2 (2)	
**Sexual maturity**			
Prepubertal	5.8 (4)	0.0 (0)	
Pubertal	79.7 (55)	100.0(9)	0.32(b)
Postpubertal	14.5 (10)	0.0 (0)	
**Nutritional status**			
Eutrophy	37.1 (26)A	11.1(1)B	
Overweight	37.7 (26)A	0.0 (0)B	0.001(b)
Obesity	25.2 (17)B	88.9 (8)A	
**Waist circumference**			
Normal	95.7 (66)	22.2(2)	<0.0001(a)
Elevated	4.3 (3)	77.8 (7)	
**Neck circumference**			
Normal	81.2 (56)	44.4 (4)	0.03(a)
Elevated	18.8 (13)	55.6 (5)	
**Systolic blood pressure classification**			
Normal	81.2 (56)	11.1 (1)	<0.0001(a)
Elevated	18.8 (13)	88.9 (8)	
**Diastolic blood pressure classification**			
Normal	89.9 (62)	22.2 (2)	<0.0001(a)
Elevated	10.1 (7)	77.8 (7)	
**Hypovitaminosis D**			

No	12.7(8)	12.5(1)	1.00[(a)]
Yes	87.3(55)	87.5(7)	
**Calcium levels**[(c)]			
Normal	100.0 (67)	100.0(9)	-
Elevated	0.0 (0)	0.0 (0)	
**Plasmatic phosphorous levels**[(c)]			
Normal	70.8(46)	50.0(4)	0.25[(a)]
Altered	29.2(19)	50.0(4)	
**Parathormone levels**[(c)]			
Normal	90.3 (56)	100.0(8)	1.00[(a)]
Altered	9.7 (6)	0.0 (0)	
**Insulin levels**[(c)]			
Normal	65.6 (40)	11.1(1)	0.003[(a)]
Elevated	34.4 (21)	88.9(8)	
**Glycemia**[(c)]			
Normal	98.5 (65)	100.0(8)	1.00[(a)]
Elevated	1.5 (1)	0.0 (0)	
**Glycated hemoglobin levels**[(c)]			
Normal	98.4 (61)	100.0(8)	1.00[(a)]
Elevated	1.6 (1)	0.0 (0)	
**Total cholesterol levels**[(c)]			
Normal	74.2 (49)	11.1(1)	0.0004[(a)]
Elevated	25.8 (17)	88.9 (8)	
**HDL levels**[(c)]			
Normal	81.8(54)	66.7 (6)	0.37[(a)]
Altered	18.2 (12)	33.3 (3)	
**Triglyceride levels**[(c)]			

Normal	47.0 (31)	11.1 (1)	0.07[(a)]
Elevated	53.0 (35)	88.9 (8)	

The results are presented as relative frequency (absolute frequency). (a) Fisher's exact test; (b) Chi-square test; (c) No information from some participants. Different letters in the column indicate significant differences between girls with and without MS (chi-square test with Bonferroni correction, $p<0.05$).
